# Object-Specific Four-Path Network for Stroke Risk Stratification of Carotid Arteries in Ultrasound Images

**DOI:** 10.1155/2022/2014349

**Published:** 2022-04-25

**Authors:** Wei Ma, Yujiao Xia, Xiaoyan Wu, Zheng Yue, Xinyao Cheng, Aaron Fenster, Mingyue Ding

**Affiliations:** ^1^Medical Ultrasound Laboratory, Department of Biomedical Engineering, College of Life Science and Technology, Huazhong University of Science and Technology, Wuhan 430074, China; ^2^College of Computer Science, South-Central University for Nationalities, Wuhan 430074, China; ^3^Key Laboratory of Molecular Biophysics of Education Ministry of China, Huazhong University of Science and Technology, Wuhan 430074, China; ^4^Department of Cardiology, Zhongnan Hospital, Wuhan University, Wuhan 430071, China; ^5^Imaging Research Laboratories, Robarts Research Institute, Western University, London, Ontario, Canada

## Abstract

Atherosclerotic carotid plaques have been shown to be closely associated with the risk of stroke. Since patients with symptomatic carotid plaques have a greater risk for stroke, stroke risk stratification based on the classification of carotid plaques into symptomatic or asymptomatic types is crucial in diagnosis, treatment planning, and medical treatment monitoring. A deep learning technique would be a good choice for implementing classification. Usually, to acquire a high-accuracy classification, a specific network architecture needs to be designed for a given classification task. In this study, we propose an object-specific four-path network (OSFP-Net) for stroke risk assessment by integrating ultrasound carotid plaques in both transverse and longitudinal sections of the bilateral carotid arteries. Each path of the OSFP-Net comprises of a feature extraction subnetwork (FE) and a feature downsampling subnetwork (FD). The FEs in the four paths use the same network structure to automatically extract features from ultrasound images of carotid plaques. The FDs use different object-specific pooling strategies for feature downsampling based on the observation that the sizes and shapes in the feature maps obtained from FEs should be different. The object-specific pooling strategies enable the network to accept arbitrarily sized carotid plaques as input and to capture a more informative context for improving the classification accuracy. Extensive experimental studies on a clinical dataset consisting of 333 subjects with 1332 carotid plaques show the superiority of our OSFP-Net against several state-of-the-art deep learning-based methods. The experimental results demonstrate better clinical agreement between the ground truth and the prediction, which indicates its great potential for use as a risk stratification and as a monitoring tool in the management of patients at risk for stroke.

## 1. Introduction

Ischemic stroke is one of the leading causes of mortality and disability worldwide, and its prevalence is increasing yearly, leading to a large financial burden on society and families [1, 2]. The prevention and management of patients at risk for ischemic stroke have become a critical issue over the past few years [3–6]. Carotid plaque is identified as one of the main sources of ischemic stroke. The rupture of unstable carotid plaques and the subsequent cascade can lead to thrombosis and subsequent cerebral emboli, which block the downstream blood vessels and result in ischemic stroke [7]. Thus, the instability of carotid plaques is related to the probability of the occurrence of stroke [8–12]. These findings have led to explorations of the factors that lead to plaque vulnerability, such as intraplaque hemorrhage and other plaque characteristics, and are of great clinical significance for identifying patients at high risk versus low risk. Furthermore, identifying patients at risk would be beneficial in assessing the effects of medical treatment and improving their management, thus, preventing stroke [13].

Ultrasound (US) imaging is a preferred modality for detecting carotid atherosclerotic plaques due to its advantages of being nonionizing, low cost, real-time imaging, and user-friendly. It is convenient to use B-mode ultrasound (BUS) for monitoring plaque regression and progression in response to medical therapy [14, 15] and evaluating the risk of atherosclerotic events [16]. Over the past decades, many studies have focused on plaque characterization by finding and quantifying carotid plaque features for the evaluation of the risk for an atherosclerotic event [17, 18]. Mathiesen et al. carried out a longitudinal population-based study and found that the total plaque area (TPA) appears to be a stronger risk predictor than intima-media thickness (IMT) for a first-ever ischemic stroke [10] [19]. With the development of three-dimensional (3D) ultrasound technology, total plaque volume (TPV), as a 3D feature of plaque, emerged [20, 21]. Wannarong et al. measured the TPV, TPA, and IMT in 349 patients and concluded that the measurement of TPV is superior to both IMT and TPA for assessment of the response to antiatherosclerotic therapy [11]. Alternative carotid plaque metrics such as large lipid cores, ulceration, and intraplaque hemorrhage are believed to be related to plaque vulnerability, and calcification is related to their stability [20, 22]. Sun et al. found that high lipid content and rupture of the fibrous cap of carotid plaques were strongly associated with systemic atherothrombotic risk, whereas high calcification content had no significant relationship with risk [23]. The echogenicity analysis of plaques can provide information on plaque composition since calcification and fibrous tissue are present as hyperechoic and other components as hypoechoic in ultrasound images [24]. Huang et al. developed a computer-aided method for identifying echolucent plaques from three types of plaques and obtained a classification accuracy of 77.46% and the area under the curve of 0.83, potentially improving the power of risk prediction of acute cerebral atherosclerotic events by ultrasonography [25].

Plaque texture features can also provide information for risk assessment, and the methods of extracting textural features from carotid plaques in ultrasound images have been widely used. Acharya et al. described a computer-aided diagnosis (CAD) system which analyzed ultrasound images and classified them into symptomatic and asymptomatic based on the textural features [26]. Then, Acharya et al. used 32 texture features along with the degree of carotid artery stenosis as a feature set in a support vector machine (SVM) classifier for the classification of symptomatic vs. asymptomatic plaques and obtained an accuracy of 90.66%, sensitivity of 83.33%, and specificity of 95.39% [27]. In 2017, Araki et al. proposed a new approach for risk assessment by calculating 16 gray-scale texture features and feeding them into a machine learning system. The mean classification accuracy for all sets of partition protocols for the automated system in the far and near carotid arterial walls were 95.08% and 93.47%, respectively [28]. Engelen et al. measured 376 samples of 3D carotid ultrasound plaques in 298 patients at baseline and a year later concluded that the changes in plaque texture were strongly predictive of atherosclerotic events [12].

All the methods mentioned above mainly use one or more hand-crafted features to train their respective models for the classification. These hand-crafted features can only describe the low-level image features, which may not represent plaque characterization comprehensively and may not identify the different carotid plaques in the grayscale ultrasound image pattern. Furthermore, the selection and combination of features from a large number of handcrafted features are time-consuming, labor-intensive, and subjective, which results in variations in the methods' accuracies.

With the evolution of novel and powerful deep learning (DL) methods, many have been applied to medical image analysis with great success in tasks such as segmentation, registration, and classification [29, 30]. Deep learning methods overcome the difficulty in manual definition and selection of features. It can automatically extract features and may mine new and high-level plaque features. Lekadir et al. proposed a deep learning-based classification method that utilized a convolutional neural network (CNN) for the automatic identification of different carotid plaque constituents, which were used for early risk estimation of atherosclerotic events [31]. They used approximately 90,000 plaque image patches with a size of 15∗15 pixels extracted from longitudinal ultrasound images of the carotid arteries as the input to the network. Experimental results showed a correlation of about 0.90 between the automatic and expert assessments of the lipid core, fibrous cap, and calcified tissue areas [31]. Kats et al. used the faster region-based convolutional neural network (Faster R-CNN) model on a small dataset of 65 images for the detection of carotid plaques in panoramic radiographs and achieved an accuracy of 83%, showing the efficiency of the Faster R-CNN algorithm in plaque detection task [32]. Furthermore, Faster R-CNN was also used by Jain et al. for the automated localization of the common carotid artery in transverse sections in B-mode ultrasound images [33]. In another study, Skandha et al. designed a computer-aided diagnosis (CADx) system consisting of three kinds of deep learning classification paradigms for cardiovascular/stroke risk stratification using carotid ultrasound-based delineated plaque [34].

However, most studies characterized carotid plaques using a single section of either the transverse or longitudinal carotid ultrasound images. The features extracted from ultrasound carotid plaques in both transverse and longitudinal sections have the potential to better represent the plaque characteristics without the need to obtain a 3D ultrasound image of carotid plaques [35]. In addition, since the degree of atherosclerosis in each of the bilateral carotid arteries is different [36], the images from bilateral carotid arteries may more comprehensively evaluate the progression of carotid atherosclerosis and more accurately classify the patients as to their risk for stroke. To this end, we propose an object-specific four-path network (OSFP-Net) to integrate carotid plaque features of two orthogonal 2D ultrasound images obtained from bilateral carotid arterial ultrasound examinations.

The OSFP-Net is comprised of four paths, which accept four carotid plaque images of the bilateral carotid arteries in transverse and longitudinal sections simultaneously as input. Each path contains a feature extraction subnetwork (FE) and a feature downsampling subnetwork (FD). The FEs use the network of the same structure to automatically extract the features from carotid plaque images. The FDs use different object-specific pooling strategies for downsampling based on the observation that the ultrasound carotid plaque images in longitudinal and transverse sections have different anatomical shapes and sizes. Since the carotid plaques are of arbitrary size and are encompassed by an approximate square in transverse ultrasound images, a spatial pyramid pooling (SPP) strategy is needed for feature downsampling [37], which is able to generate a fixed-length representation regardless of the input size. Similarly, the ultrasound carotid plaques in longitudinal sections are not only of arbitrary sizes but also appear elongated. Therefore, the multilevel strip pooling (MSP) strategy was adopted [38], which can not only accept inputs of arbitrary sizes but also can enlarge the receptive field to obtain a long-range informative context. Both object-specific pooling strategies are lightweight and can serve as efficient add-on blocks to be plugged into the backbone networks to learn more complementary information of plaque images from different sections, which may help to identify the differences of carotid plaques in patients that pose a risk for stroke [39]. As a consequence, the proposed method has potential superior performance to several popular DL-based methods for classification.

The contributions of this work are summarized as follows:

1. We propose an OSFP-Net, which consists of four paths for simultaneous acceptance as input of four arbitrarily sized plaques in bilateral carotid ultrasound images in both transverse and longitudinal sections. The FE in each path automatically extracts features for the classification and is designed to mimic the radiologist in clinical practice by performing a more comprehensive observation of the carotid plaques than just one section used in other studies. In addition, since the elevational and in-plane resolutions of the ultrasound images are different, the two orthogonal images of carotid plaques provide complementary morphological information for the feature representation for carotid plaque classification
2. In the FDs, we adopt different object-specific pooling strategies for the features downsampling based on the consideration that the sizes in the feature maps obtained from the four FEs should be different theoretically. SPP is suitable for feature downsampling of carotid plaques in a transverse section, while MSP is adapted for those in a longitudinal section. Both of these can accept carotid plaques of arbitrary sizes and enlarge the receptive field so that they capture a more informative context to improve the classification performance
3. We experimentally validated our approach demonstrating that OSFP-Net is able to achieve higher accuracy for the classification of symptomatic and asymptomatic subjects on the collected dataset as an indication of classification of patients with unstable and stable plaques. The experimental results demonstrated that OSFP-Net compares favorably to the baseline and existing popular CNNs

In this paper, Section 2 presents the patient demographics, data acquisition, the proposed network details, the experimental setup, and the classification metrics.  Section 3 presents the experimental results, the discussion is in Section 4, and the study concludes in Section 5.

## 2. Materials and Methods

### 2.1. Patient Demographics, Data Acquisition, and Preprocessing

**Figure 1:**
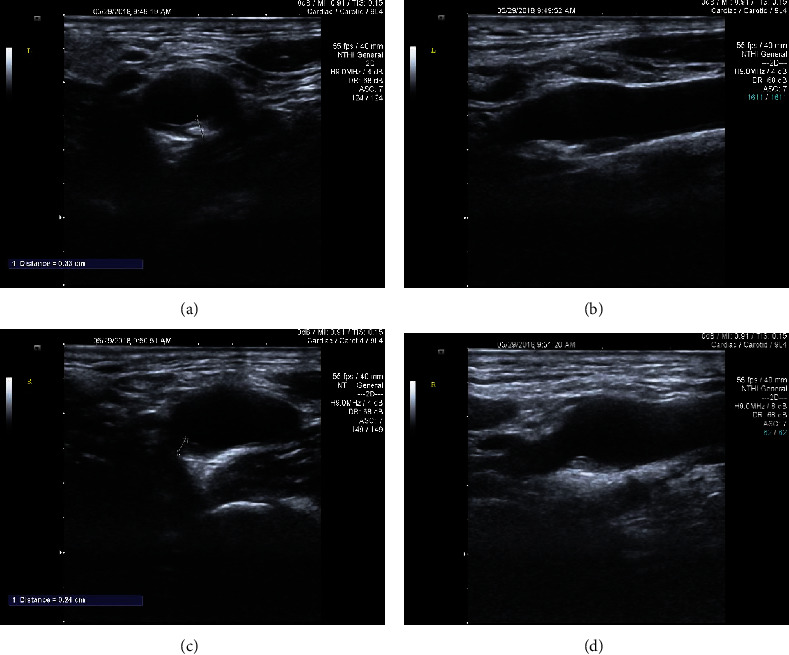
Four different ultrasound images of plaques from a patient. (a) Transverse section of a plaque in the left carotid artery; (b) longitudinal section of a plaque in the left carotid artery; (c) transverse section of a plaque in the right carotid artery; (d) longitudinal section of a plaque in the right carotid artery.

Patients with carotid plaques in the study were imaged in the Department of Neurology and the Department of Cardiology at ZhongNan Hospital, Wuhan University, China. A Siemens ACUSON SC2000 ultrasound imaging system with a 9L4 linear probe (Siemens, Berlin, Germany) was used to acquire the carotid ultrasound images by two qualified, experienced physicians (X.C. and X.W have 35 and 13 years of experience, respectively, the two coauthors of this paper). Ultrasound imaging of the carotid arteries involved scanning upward from the patient's clavicle to detect plaques in the common carotid artery, carotid sinus, internal carotid artery, and external carotid artery. The transverse and longitudinal images of the largest plaque area [10] from the bilateral carotid arteries were acquired, resulting in four images for each patient (Figure 1). The study was approved by the Hospital Institutional Review Board, and each participating patient was consented. For each patient, the Framingham clinical data [40] was also collected, including the patient's gender, age, body mass index (BMI), blood pressure, blood lipids, smoking status, and the history of atherosclerotic events (Table 1).

Although papers have been published on plaque vulnerability using various methods by identifying plaque composition [31, 32], it is not possible to use an independent assessment of the risk a carotid plaque poses without following patients with carotid atherosclerosis for many years and scoring those who experienced transient ischemic attacks (TIA) or strokes that are attributed to carotid plaques. As an alternative, we used the ultrasound images with carotid plaques of patients who experienced a cerebrovascular event and those who did not as surrogate biomarkers of patients with and without vulnerable plaques. Since atherosclerosis is a systemic disease, those patients who suffered cerebrovascular events may be due to plaque disruption in other vessels and most probably have vulnerable plaques in the carotid arteries [41].

Thus, in our study, symptomatic patients were included if they had experienced a TIA or ischemic stroke, while asymptomatic patients were included if they had carotid plaques but did not suffer a stroke or TIA. In total, 333 patients, including 117 patients with atherosclerotic events and 216 event-free patients, were analyzed in the study, where four carotid ultrasound images (the largest plaques of bilateral carotid arteries in transverse and longitudinal sections) were acquired for each patient generating a total of 1332 images.

### 2.2. Plaque Segmentation and Data Augmentation

**Figure 2:**
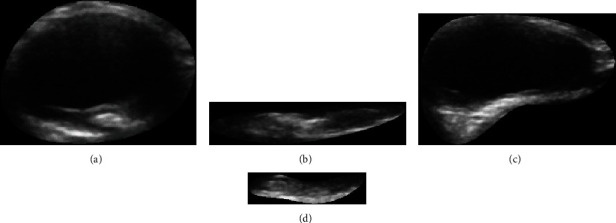
ROI for plaques in the bilateral carotid artery images in both transverse and longitudinal sections. (a) ROI of the left transverse plaque image corresponding to Figure 1(a); (b) ROI of the left longitudinal plaque image corresponding to Figure 1(b); (c) ROI of the right transverse longitudinal image corresponding to Figure 1(c); (d) ROI of the right longitudinal plaque image corresponding to Figure 1(d).

Region-of-interest (ROIs) was manually selected in the transverse sections by removing the background beyond the carotid adventitia of the vessels as shown in Figures 2(a) and 2(c), and in longitudinal sections by selecting the ROI that encompassed the plaque as shown in Figures 2(b) and 2(d). It should be noted that an automatic segmentation method for carotid plaques has been studied by another member of our laboratory [42]. Because segmentation is not the focus of this study, the manual segmentation results were used as the ROIs. The ROIs of all images had arbitrary sizes for use in the training and testing.

To increase the sample size of our collected dataset, data augmentation techniques were used to obtain additional images [43]. The acquired dataset was augmented using scaling and flipping operations, by scaling factors of 0.8, 0.9, 1.1, and 1.2 (4-fold increase), and flipping horizontally and vertically (2-fold increase), resulting in a factor of 7 image augmentation, giving a total of 9324 images. This allows an investigation of the classification performance based on training the proposed network with or without augmentation.

### 2.3. Object-Specific Four-Path Network (OSFP-Net)

**Figure 3:**
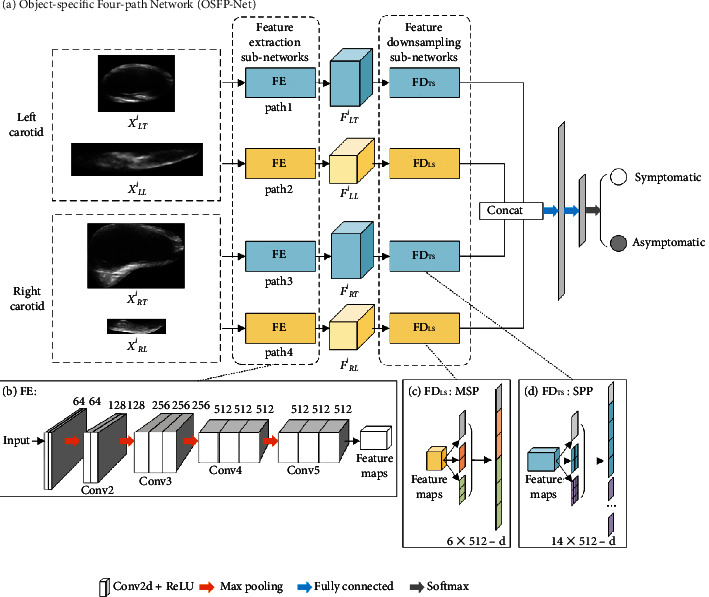
Architecture of the object-specific four-path network (OSFP-Net). (a) The OSFP-Net is comprised of four paths for inputs of the bilateral carotid plaque images in both the transverse and longitudinal sections. Each path contains a feature extraction subnetwork (FE) and a feature downsampling subnetwork (FD). (b) The FE employs the same 5 convolutional and pooling blocks as VGG16, which are mainly used for image feature extraction. (c) The FD_LS_ applies a multilevel strip pooling (MSP) strategy for the carotid plaques in the longitudinal section. (d) The FD_TS_ employs a spatial pyramid pooling (SPP) strategy for the carotid plaques in the transverse section.

Figure 3(a) shows the architecture of OSFP-Net, which was used for feature extraction and downsampling of the carotid plaques from the two orthogonal views of the bilateral carotid arteries ultrasound images. Each path in the OSFP-Net was composed of two sub-networks: a feature extraction subnetwork and an object-specific feature downsampling subnetwork. As shown in Figures 3(c) and 3(d), FD_TS_ was used to downsample the feature maps obtained from the carotid plaque images in the transverse sections from both sides of the patient, while FD_LS_ was used to downsample the feature maps obtained from the carotid plaque images in longitudinal sections from both sides of the patient. In the forward propagation, the four carotid plaque images of arbitrary sizes are fed into the FEs for feature extraction and four groups of feature maps with different sizes are obtained. The outputs of the FEs are then connected to the FDs to perform feature downsampling. Each FD produces a vector of fixed length. These vectors are then concatenated and fed into the fully connected layers for classification. Of note, the feature maps extracted from OSBP-Net are more distinct and comprehensive compared with those from a single path CNN, thus providing a superior ability of discriminative feature learning for the classification of carotid plaques.

In addition, as we used a relatively small sample dataset size, we used the publicly available weights for the VGG16, which was trained against the ILSVRC12 challenge data set, and then finetuned through transfer learning [44] for use in our study. A dropout layer [45] was added to the network before the last fully connected layer and the feed-forward operation in the network with dropout is shown in equations (1)–(4). Here, the Bernoulli function was used to randomly generate a vector of 0 or 1. *z*^(*l*)^ denotes the vector of the inputs into layer *l*, and *y*^(*l*)^ denotes the vector of outputs from layer *l*. *w*^(*l*)^ and *b*^(*l*)^ are the weights and biases at layer *l* [45].

(1) $$\begin{array}{c} r_{j}^{\left(l\right)}∼\text{Bernoulli}\left(p\right), \end{array}$$

(2) $$\begin{array}{c} {\tilde{y}}^{\left(l\right)}=r^{\left(l\right)}*y^{\left(l\right)}, \end{array}$$

(3) $$\begin{array}{c} z_{i}^{\left(l+1\right)}=w_{i}^{\left(l+1\right)}{\tilde{y}}^{l}+b_{i}^{\left(l+1\right)}, \end{array}$$

(4) $$\begin{array}{c} y_{i}^{\left(l+1\right)}=f\left(z_{i}^{\left(l+1\right)}\right). \end{array}$$

#### 2.3.1. Feature Extraction Subnetworks

As shown in Figure 3(b), the FEs in the four paths employ the same architecture, which is identical to the convolution and pooling blocks as in VGG16, except for the pooling layer after the last convolution layer. Each block has multiple convolution layers (with rectified linear unit (ReLU) activation), which uses 3 × 3 filters with strides and paddings of 1, along with 2 × 2 max-pooling layers with strides of 2. The convolution layers operate in a sliding window manner to perform feature extraction on the input carotid plaque images of arbitrary sizes and generate feature maps of any size. The inputs for each of the four subnetworks are four carotid plaque images of the *i*th subject: the left carotid images in the transverse and longitudinal sections (*X*_*LT*_^*i*^, *X*_*LL*_^*i*^) and the right carotid images in the transverse and longitudinal sections (*X*_*RT*_^*i*^, *X*_*RL*_^*i*^). Here, the superscript *i* denotes the *i*th subject, the first subscripts “*L*” and “*R*” represent the left and right carotid arteries, and the second subscripts “*T*” and “*L*” represent the transverse and longitudinal sections. Such a strategy enables the FEs to extract multiview features from four input images of the carotid plaques simultaneously and therefore helps to improve the prediction accuracy of the OSBP-Net. Generally, the image sizes of the bilateral carotid plaques in the longitudinal and transverse sections are different. Therefore, the sizes of the feature maps extracted by FEs from the four input images are also different.

#### 2.3.2. Feature Downsampling Subnetworks

As illustrated in Figures 3(c) and 3(d), FD is a composite layer of multilevel pooling. The pooling strategy of this layer positively impacts the performance of the network, especially for objects of arbitrary sizes. As mentioned above, the feature maps extracted from the four input images of arbitrary sizes by the FEs are also arbitrarily sized. Such observation motivates us to use different pooling strategies in the FDs of different paths. As the ROIs are similar to squares for the transverse-sectional carotid images in paths 1 and 3, the SPP is needed to perform feature downsampling and generate a fixed-length representation regardless of the sizes of the feature maps. For longitudinal-sectional carotid plaque images, the ROIs are approximately long-strips. To ensure the FDs enlarge the receptive field and acquire more long-range context, an MSP module is required.

Let the size of the *k* feature maps extracted from the bilateral transverse-sectional carotid plaque images in paths 1 and 3 using the SPP module be *H*_*LT*_^*i*^ × *W*_*LT*_^*i*^ and *H*_*RT*_^*i*^ × *W*_*RT*_^*i*^. The output vectors *V*_*LT*_ and *V*_*RT*_ obtained in FDs using *j*-level SPP module of pools (*a*_*n*_ × *a*_*n*_, *n* = 1, 2, ⋯*j*) can be written as follows:

(5) $$\begin{array}{c} V_{LT}=V_{RT}=k\overset{j}{\underset{n=1}{\sum }}a_{n}^{2}. \end{array}$$

Similarly, let the size of the *k* feature maps extracted from bilateral longitudinal-sectional carotid plaque images in paths 1 and 3 using the MSP module be *H*_*LL*_^*i*^ × *W*_*LL*_^*i*^ and *H*_*RL*_^*i*^ × *W*_*RL*_^*i*^. The output vectors *V*_*LT*_ and *V*_*RT*_ obtained in FDs using the *j*-level MSP module of strips (*a*_*n*_ × *b*_*n*_, *n* = 1, 2, ⋯*j*) can be calculated as follows:

(6) $$\begin{array}{c} V_{LL}=V_{RL}=k\overset{j}{\underset{n=1}{\sum }}a_{n}\times b_{n}. \end{array}$$

The meanings of superscripts and subscripts are the same as those described in 2.3.1. Details of the calculation method are given in [38]. In the training phase, we adopted different pooling settings and found that 3-level SPP module of pools (1 × 1, 2 × 2, 3 × 3) and 3-level MSP module of strips (1 × 1, 2 × 1, 3 × 1) result in the best prediction. The settings and outputs are indicated in Table 2.

### 2.4. Experimental Setup

We used an open-source deep learning framework, PyTorch, for training and testing the proposed network and popular CNNs for comparison purposes. All training and testing procedures were performed on an Ubuntu 64-bit desktop personal computer with an Intel Core I9-10900K central processing unit (CPU) and 32 GB of random-access memory. An NVIDIA RTX 2080 Ti graphical processing unit (GPU) with CUDA 10.1 was used for acceleration.

The cross-entropy function was used as the cost function, and the stochastic gradient descent (SGD) optimizer was adopted to minimize the cost function [46]. The number of iterations was 30, the momentum was 0.9, and the learning rate was set to 0.001, which was reduced by a factor of 10 after every 6 iterations.

During the training and testing phases, we used batch data to train the network. The batch data needed to be consistent in all dimensions because the batch array was required to be converted into a tensor during the training and testing phases. Consequently, the batch size was set to 1 when using OSFP-Net accepted images with arbitrary sizes as inputs.

### 2.5. Evaluation Metrics

In this paper, the aim was to identify the plaque differences between the patients who experienced atherosclerotic events or those who were event-free, which is a binary classification problem. Thus, we used the following five common classification evaluation metrics to evaluate the classification performance of OSFP-Net.

(7) $$\begin{array}{c} \text{accuracy}=\frac{\text{TP}+\text{TN}}{\text{TP}+\text{FP}+\text{TN}+\text{FN}}, \end{array}$$

(8) $$\begin{array}{c} \text{sensitivity}=\frac{\text{TP}}{\text{TP}+\text{FN}}, \end{array}$$

(9) $$\begin{array}{c} \text{specificity}=\frac{\text{TN}}{\text{TN}+\text{FP}}, \end{array}$$

(10) $$\begin{array}{c} \text{precision}=\frac{\text{TP}}{\text{TP}+\text{FP}}, \end{array}$$

(11) $$\begin{array}{c} F1-\text{score}=\frac{2\times \text{precision}\times \text{recall}}{\text{precision}+\text{recall}}, \end{array}$$

where TP, FP, TN, and FN represent the numbers of true-positive, false-positive, true-negative, and false-negative cases, respectively. Sensitivity measures the ability to correctly recognize positive cases, while specificity indicates the ability to correctly classify negative cases. Precision denotes the proportion of positive cases that were classified as positive cases, and the *F*1-score represents the harmonic average of precision and recall and is typically used for the optimization of a model towards either precision or recall.

In addition, a receiver operating characteristic (ROC) curve [47] was generated to further analyze the classification performance of the proposed OSFP-Net by determining the false positive rate (FPR = 1 − specificity) and the true positive rate (TPR = sensitivity). The area under the ROC curve (AUC) [48] was then calculated to provide the evaluation metric. Since the proposed algorithm performance may be higher or lower than the means obtained by the other methods, we used the two-sided *T*-test to test whether our method is statistically significantly different from the other methods [49]. Furthermore, we used the paired *T*-test for the analysis since we compared the metrics obtained for the same plaque generated by the different methods [50]. A Holm-Bonferroni correction [51] was applied for adjusting the *p* values when multiple *T*-tests were used. The Holm-Bonferroni correction is a commonly used version of the Bonferroni correction method that is less conservative but did not change the conclusion compared to the use of the Hochberg test [52]. The corrected *p* value for the *k*th-test, denoted *p*_*k*_ is computed as

(12) $$\begin{array}{c} p_{k}=\left(N-k+1\right)p, \end{array}$$

when there are *N* comparisons. If the *p*_*k*_ is less than the given significance level *α*, which is 0.05, it indicates that the results of the two methods are significantly different.

### 2.6. Experimental Protocol

A cross-validation (CV) paradigm that uses the K5 protocol (80% training and 20% testing) was employed to ensure the reliability of the results and comprehensive evaluation. The 333 patients in the dataset were randomly and equally divided into five subsets, ensuring the training and testing subsets did not overlap. The five subsets were obtained by using the following method. First, we numbered the 333 patients and set the number of patients in each subset to be in the range from 65 to 70. Then, we used a random seed to generate five numbers within the range, such that the sum of the five numbers was 333. Based on the five numbers, we randomly sampled patients from the 333 samples to obtain the five subsets. The numbers were 66, 66, 66, 66, and 69, resulting in five groups of patients that did not overlap, and the sample sizes were relatively balanced. For each experiment, four subsets were used for training, and one subset was used for testing. For the classification evaluation indicator (such as accuracy), the five values generated by the 5-fold cross-validation were then averaged. Among the five experiments, the best model parameters obtained during the 5-fold cross-validation were used to construct the proposed OSFP-Net. Note that the average value of the 5-fold cross-validation experiments was used in the evaluation of the metrics.

## 3. Results and Discussion

### 3.1. Effectiveness of the OSFP-Net

**Figure 4:**
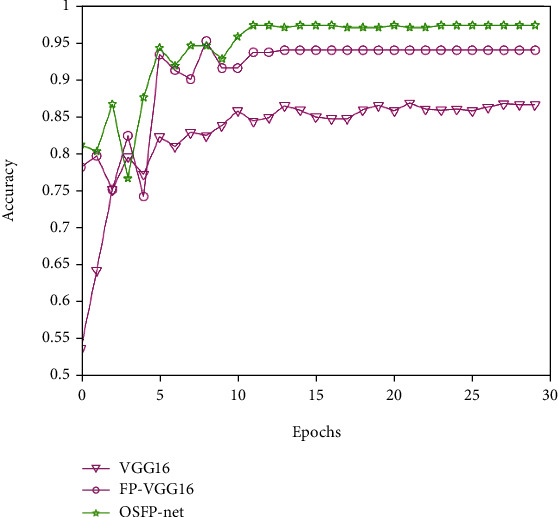
Accuracy comparison between the baseline VGG16, FP-VGG16, and OSFP-Net as a function of network epochs.

The first experiment was conducted to verify the classification performance of OSFP-Net using the four plaque images simultaneously as inputs of the transverse and longitudinal bilateral carotid sections. We compared the predictions of the baseline one-path VGG16, four-path VGG16 (FP-VGG16), and the OSFP-Net. The sample size and data set partitioning are shown in Table 3. The experimental results are illustrated in Figure 4, which shows that the OSFP-Net accurately classified the two carotid plaque types and achieved superior performance (ACC: 97.3%) over the baseline VGG16 (ACC: 86.6%) and FP-VGG16 (ACC: 93.9%). As shown in Table 4, the OSFP-Net achieved an overall classification sensitivity of 96.2%, specificity of 97.6%, precision of 95.8%, and *F*1-score of 95.9%. Compared with the baseline VGG16, the OSFP-Net outperforms it in terms of sensitivity (14.4% improvement), specificity (9.6% improvement), precision (17.6% improvement), and *F*1-score (16.1% improvement). The comparison with FP-VGG16 showed that although the specificity and accuracy obtained by OSFP-Net in fold1 and fold5 are slightly lower than FP-VGG16, the sensitivity and *F*1-score are higher than FP-VGG16. Moreover, in fold2, fold3, and fold4, the performance metrics obtained by OSFP-Net were superior to FP-VGG16 resulting in an overall performance of OSFP-Net outperforming FP-VGG16. These results demonstrate that the OSFP-Net with the integration of the four paths improves the classification performance and shows that object-specific pooling modules are a powerful supplement for the network.

### 3.2. Effect of Sample Size on Performance Using OSFP-Net

This experiment evaluated the effect of sample size on the classification performance for risk assessment using OSFP-Net. In this work, the sample size was increased using data augmentation, which included image scaling and flipping. This protocol made use of the optimal kernel, which was obtained in the first experiment (Section 3.1). This paradigm for increased sample size using data augmentation is repeated for each fold of 5-fold cross-validation. For ease of comparison, we only increased the samples size of the training set, and the samples and quantity of the testing set were the same as the first experiment.

**Figure 5:**
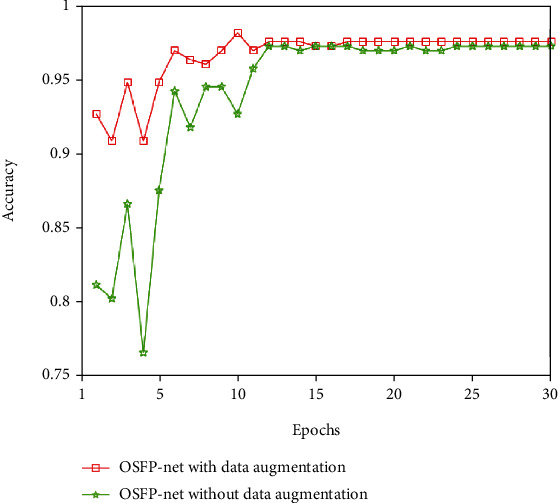
Accuracy comparison using the dataset with or without data augmentation.

As shown in Figure 5, when the training set was augmented with data augmentation, the accuracy obtained on the testing set is higher than that without data augmentation before the 12th epoch, but there is no significant difference after the 12th epoch. In both cases, the accuracy exceeded 95%.

Table 5 shows the classification metrics obtained on the testing set using OSFP-Net with or without data augmentation. The results of the *T*-tests showed that there were no statistically significant differences between the results when the training was performed with and without data augmentation, indicating that OSFP-Net has good learning potential and classification performance on a small sample size without data augmentation.

### 3.3. Comparison with the State-of-the-Art Classification Networks

**Figure 6:**
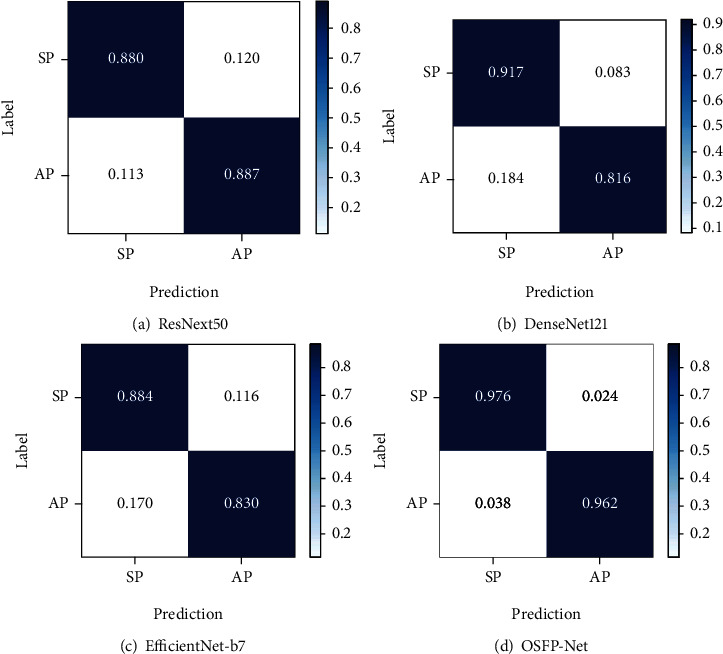
Confusion matrices of the compared networks for the classification of carotid plaques. SP and AP represent symptomatic and asymptomatic patients, respectively.

**Figure 7:**
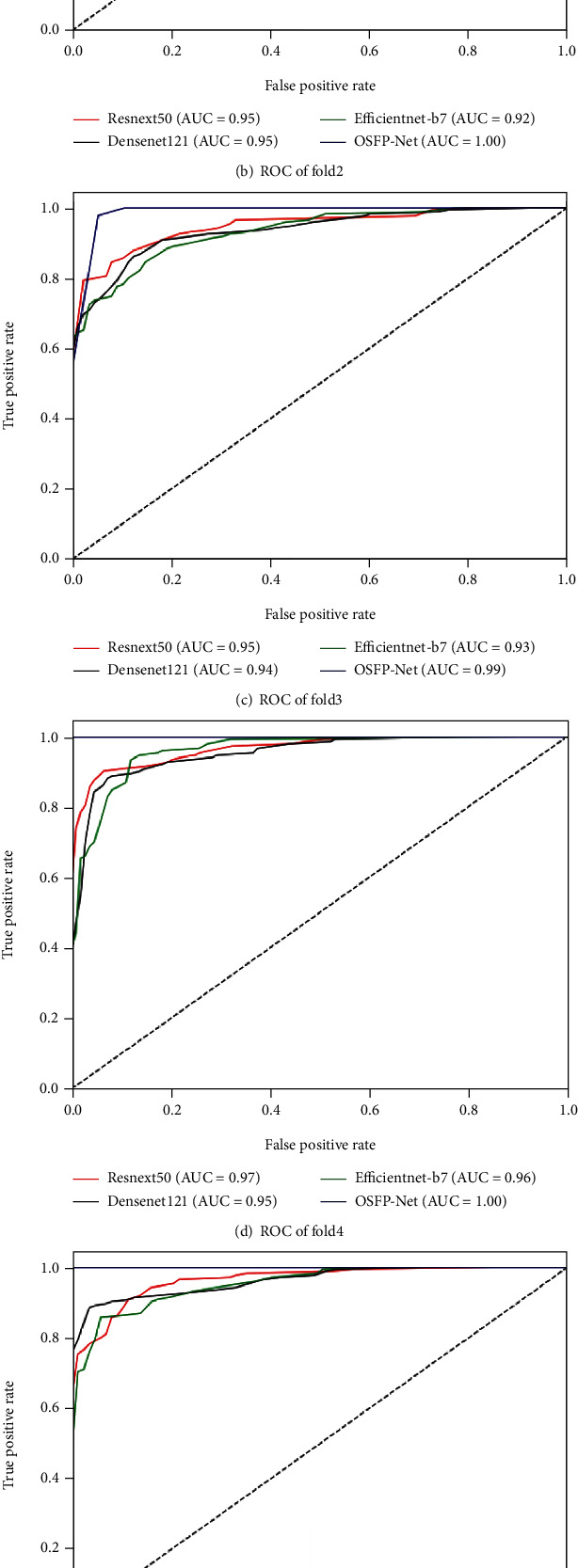
ROC curves for discriminating symptomatic and asymptomatic patients based on carotid plaque images for all the compared networks using 5-fold cross-validation on the collected dataset. AUC: area under the curve.

Table 6 and Figures 6 and 7 show the results of the comparison of the proposed approach with previous state-of-the-art classification methods. As shown in Table 6, our approach using OSFP-Net outperforms all of the well-known classification methods including ResNext50 [53], DenseNet121 [54], and EfficientNet-b7 [55] in all metrics. As shown in Table 6, OSFP-Net obtained better performance than ResNext50 in the term of accuracy (8.9% improvement), sensitivity (7.5% improvement), specificity (9.6% improvement), precision (16.1% improvement), and *F*1-score (12.0% improvement), than DenseNet121 in terms of accuracy (9.1% improvement), sensitivity (14.6% improvement), specificity (5.9% improvement), precision (12.2% improvement), and *F*1-score (13.4% improvement), and than EfficientNet-b7 in the term of accuracy (10.8% improvement), sensitivity (13.2% improvement), specificity (9.2% improvement), precision (17.1% improvement), and *F*1-score(15.2% improvement). *T*-tests comparing the metrics generated by OSFP-Net to the other methods showed that there were statistically significant differences.

Figure 6 shows the confusion matrices of ResNext50 [53], DenseNet121 [54], EfficientNet-b7 [55], and OSFP-Net for the classification of the symptomatic and asymptomatic patients. It is apparent that the proposed OSFP-Net provided the best classification rates for the two types of patients. For the symptomatic patients, our proposed network achieves an accuracy of 0.976, while the highest accuracy among popular CNNs is 0.917 obtained by DenseNet121. The accuracy of ResNext50 and EfficientNet-b7 in this category is lower than 0.900. Although DenseNet121 achieved the second-highest classification rate of 0.917 for symptomatic patients, it performed poorly in the classification of asymptomatic patients, which were misclassified at a rate of 0.184 symptomatic patients.

The ROC curves for OSFP-Net and all compared networks are given in Figure 7. The ROC analysis showed that area under the curve (AUC) obtained by OSFP-Net was 0.99 on fold3 and was equal to 1 on other folds, which were higher than those of ResNext50, DenseNet121, and EfficientNet-b7. The AUC of the average ROC obtained by OSFP-Net is 1, which indicates good precision of OSFP-Net for the classification of carotid plaques.

### 3.4. Discussion

The accurate and objective classifications of carotid plaques provide important information for stroke risk assessment and can help to plan optimal treatment strategies [56, 57]. In this study, a novel classification method, OSFP-Net, was proposed to classify carotid plaques in patients who are symptomatic and asymptomatic, which is an indication of patients who do not have or do have vulnerable plaques. Although asymptomatic patients may progress to have vulnerable plaques and become symptomatic, at the time of imaging, their plaques appeared to be stable. Thus, periodic imaging of asymptomatic patients with carotid plaques may identify when their plaques become vulnerable. As well, periodic imaging of patients with vulnerable plaques who are being treated medical (e.g., statins and diet) may help to identify if their plaques become less dangerous [57].

Our method is based on and extends the commonly used clinical 2D ultrasound imaging and examination method of the bilateral carotid plaques in both the transverse and longitudinal sections. Current published classification research on carotid ultrasound images only analyzed plaques from a single section, either in a transverse or longitudinal section. We explored using four images of plaques of arbitrary sizes from the bilateral carotid arteries in both sections as simultaneous inputs and employed two different object-specific pooling strategies to perform feature downsampling, to provide a more comprehensive and informative feature representation to boost the accuracy without the need for 3D US imaging. The results demonstrate that our proposed method outperforms VGG16, FP-VGG16, ResNext50, DenseNet121, and EfficientNet-b7 with a mean test accuracy of 97.3% on the collected dataset when the total sample size was the same. Hence, this method may be used as a computer-aided assessment tool to help physicians assess the risk of stroke and the effectiveness of medical management. Furthermore, it should be noted that the performance achieved on the dataset with a small sample size is similar to that obtained by increasing the sample size through data augmentation. This indicates that the proposed method has great potential in feature learning and representation to improve classification performance on a small sample size, which is of significance for medical image classification when large datasets are not available.

Although high classification accuracy, as well as sensitivity, specificity, precision, and *F*1-score are reached in our experiment, we acknowledge a few limitations that warrant subsequent follow-up work. First, a key limitation is that the ground truth was determined by clinicians using prior history of symptoms and physical examination. However, in some cases, the symptoms may be caused by plaques in an area other than the carotid artery. Since atherosclerosis is a systemic disease, patients with unstable plaques in other vessels most likely have vulnerable plaques in the carotid arteries [41]. Nonetheless, some labeled images belonging to symptomatic patients may have been mislabeled and should have been labeled as asymptomatic leading to errors in the ground truth determination, which will result in a decrease in the classification accuracy. Second, we note that the carotid plaque images in our data set were obtained only at a one point in time, which cannot reflect the changes and regression/progression of carotid plaques. Thus, it would be important to collect carotid images and clinical information at multiple time points during follow-up sessions to provide a more accurate dataset of classified plaques and make it available to investigators to develop image-based prediction tools for the identification of risk for stroke. Third, for our study, we collected images of the largest plaque in each of the bilateral carotid arteries of each patient. If the patient has multiple plaques, further improvement on the proposed model will be required to flexibly accept variable numbers of images of plaques. Finally, in our work, since patients are routinely imaged with carotid ultrasound, we only explored whether the simple imaging of the carotid plaques can distinguish between symptomatic and asymptomatic patients as a step to provide information on vulnerable plaques. Although our proposed method has achieved satisfactory results, it may be better to combine feature representation of carotid plaques in ultrasound images with the patients' clinical information, such as Framingham clinical data [40] as listed in Table 1. These improvements remain to be further addressed in follow-up studies.

## 4. Conclusions

In this paper, we proposed a novel object-specific four path network for the classification of carotid plaques in ultrasound images to aid in the stratification of patients at risk for ischemic stroke. The proposed network simultaneously accepts four plaque images of arbitrary sizes from bilateral carotid arteries in both transverse and longitudinal sections as inputs, which captures a more comprehensive and informative feature representation to boost the performance of classification. A 5-fold cross-validation was used to evaluate the effectiveness of our network on a collected clinical dataset. The experimental results demonstrated that our network is more effective and outperforms such popular networks as ResNext50, DenseNet121, and EfficientNet-b7 in terms of accuracy, sensitivity, specificity, precision, and *F*1-score. Thus, our network may potentially assist clinicians in using a more objective risk assessment metric and monitoring tool to aid in the assessment of the risk for cerebrovascular events.

## Figures and Tables

**Table 1:** Patient demographics and imaging parameters.

Variable	Mean ± SD/Num (PCT)	Range	Count
Sex, %			333
Male	204 (61.3)		
Female	129 (38.7)		
Age, years			
All	69 ± 11	35–99	333
Male	68 ± 11	35–99	204
Female	71 ± 4	45–95	129
Body mass index, kg/m^2^	22.9 ± 3.4	18.2–35.4	106
Blood pressure, mm Hg			
Systolic	137 ± 15	76–203	333
Diastolic	78 ± 2	43–140	333
Laboratory values, mmol/L			
Total cholesterol	4.26 ± 0.62	1.71–12.03	333
Low-density lipoprotein cholesterol	2.42 ± 0.19	0.81–7.76	333
High-density lipoprotein cholesterol	1.01 ± 0.21	0.09–3.83	333
Triglycerides	1.76 ± 0.29	0.29–18.58	333
Risk factors, %			
Hypertension	218 (65.5)		333
Hyperlipidemia	58 (20.4)		285
Diabetes	93 (31.3)		297
Ever smoked	99 (43.0)		230
FRS risk, %			333
<10	36 (10.8)		
10-20	144 (43.2)		
>20	153 (45.9)		
Imaging parameters			
Ultrasound system manufacturer	Siemens		
System model	SC2000		
Ultrasound probe	9 L5		
Vessels images	Common, internal, external carotid arteries		

**Table 2:** Different settings and outputs in FD modules. k refers to the number of feature maps.

Paths	Input image	FDs				Total strips/bins	Outputs
		Module	1st-level	2nd-level	3rd-level		
1	LT	SPP	1 × 1	2 × 2	3 × 3	14 bins	14 *k*
2	LL	MSP	1 × 1	2 × 1	3 × 1	6 strips	6 *k*
3	RT	SPP	1 × 1	2 × 2	3 × 3	14 bins	14 *k*
4	RL	MSP	1 × 1	2 × 1	3 × 1	6 strips	6 *k*

**Table 3:** Comparison of sample size and data set partitioning among the baseline VGG16, FP-VGG16, and OSFP-Net.

Method	Path	Plaques	No. of input	No. of samples	Training set	Testing set
VGG16	One	1332	1	1332	1068	264
FP-VGG16	Four	1332	4	333	267	66
OSFP-Net	Four	1332	4	333	267	66

**Table 4:** Sensitivity, specificity, precision, and *F*1-score comparisons between the baseline VGG16, FP-VGG16, and OSFP-Net. The best results are highlighted in bold. The listed metrics were obtained on the test dataset.

Fold	Method	Metrics (%)			
		SEN	SPE	PRE	*F*1-score
1	VGG16	79.5	83.0	66.0	72.1
	FP-VGG16	87.5	**100.0**	**100.0**	93.3
	OSFP-Net	**95.8**	95.2	92.0	**93.9**
2	VGG16	74.0	85.5	75.5	74.7
	FP-VGG16	80.0	95.7	88.9	84.2
	OSFP-Net	**95.0**	**95.7**	**90.5**	**92.7**
3	VGG16	81.6	90.5	83.3	82.5
	FP-VGG16	80.0	100.0	100.0	88.9
	OSFP-Net	**90.0**	**100.0**	**100.0**	**94.7**
4	VGG16	84.7	89.2	86.2	85.5
	FP-VGG16	95.7	90.7	84.6	89.8
	OSFP-Net	**100.0**	**100.0**	**100.0**	**100.0**
5	VGG16	88.9	91.8	80.0	84.2
	FP-VGG16	93.1	**100.0**	**100.0**	96.4
	OSFP-Net	**100.0**	97.3	96.7	**98.3**
Avg. ±Std.	VGG16	81.8 ± 5.0	88.0 ± 3.3	78.2 ± 7.1	79.8 ± 5.4
	FP-VGG16	88.4 ± 6.8	97.3 ± 3.7	94.7 ± 6.6	91.2 ± 4.7
	OSFP-Net	**96.2 ± 3.7**	**97.6 ± 2.0**	**95.8 ± 4.0**	**95.9 ± 2.8**

**Table 5:** Classification metrics were obtained on the testing set using OSFP-Net with or without data augmentation on the training set. The *p* value for the *T*-tests is shown in the bracket. DA√ = with augmentation, DA × = no augmentation.

DA	Sensitivity (%)	Specificity (%)	Precision (%)	*F*1-score (%)
√	94.5 (*p* = 0.52)	99.1 (*p* = 0.25)	98.1 (*p* = 0.35)	96.2 (*p* = 0.87)
×	96.2	97.6	95.8	95.9

**Table 6:** The comparative accuracy, sensitivity, specificity, precision, and *F*1-score results of the proposed OSFP-Net and other well-known classification methods. The *p* value for the statistical *T*-test is shown in the brackets.

Methods	Accuracy (%)	Sensitivity (%)	Specificity (%)	Precision (%)	*F*1-score (%)
ResNext50	88.4 (1.8E-08)	88.7 (0.026)	88.0 (0.0002)	79.7 (0.0001)	83.9 (0.0002)
DenseNet121	88.2 (2.8E-09)	81.6 (0.001)	91.7 (0.0008)	83.6 (0.002)	82.5 (0.0002)
EfficientNet-b7	86.5 (1.2E-10)	83.0 (0.0009)	88.4 (7.8E-05)	78.7 (0.0007)	80.7 (0.0002)
OSFP-Net	97.3	96.2	97.6	95.8	95.9

## Data Availability

The data used to support the findings of this study are available from the corresponding author upon request.

## References

[1] World Health Organization (2018). *Cardiovascular Diseases*.

[2] Feigin L. V., Stark B. A., Johnson C. O. (2021). Global, regional, and national burden of stroke and its risk factors, 1990-2019: a systematic analysis for the Global Burden of Disease Study 2019. *The Lancet Neurology*.

[3] Media H. (2018). *Types of Strokes: Causes, Symptoms, and Treatments*.

[4] Uchiyama S., Ishizuka N., Shimada K. (2016). Aspirin for stroke prevention in elderly patients with vascular risk factors. *Stroke*.

[5] Salinas J., Schwamm L. H. (2017). Behavioral interventions for stroke prevention. *Stroke*.

[6] Krishnamurthi R., Hale L., Barker-Collo S. (2019). Mobile technology for primary stroke prevention. *Stroke*.

[7] Sallustio F., Samà D., Mascolo A. P., Marrama F., Fresilli M., Diomedi M. (2020). Clinical worsening despite intravenous thrombolysis in acute ischemic stroke secondary to carotid plaque rupture. *Journal of Thrombosis and Thrombolysis*.

[8] Hansson G. K., Libby P., Tabas I. (2015). Inflammation and plaque vulnerability. *Journal of Internal Medicine*.

[9] Gupta A., Baradaran H., Schweitzer A. D. (2013). Carotid plaque MRI and stroke risk. *Stroke*.

[10] Mathiesen E. B., Johnsen S. H., Wilsgaard T., Bønaa K. H., Løchen M. L., Njølstad I. (2011). Carotid plaque area and intima-media thickness in prediction of first-ever ischemic stroke. *Stroke*.

[11] Wannarong T., Parraga G., Buchanan D. (2013). Progression of carotid plaque volume predicts cardiovascular events. *Stroke*.

[12] van Engelen A., Wannarong T., Parraga G. (2014). Three-dimensional carotid ultrasound plaque texture predicts vascular events. *Stroke*.

[13] Roy Cardinal M. H., Heusinkveld M. H. G., Qin Z. (2017). Carotid artery plaque vulnerability assessment using noninvasive ultrasound elastography: validation with MRI. *American Journal of Roentgenology*.

[14] Kasliwal R., Kaushik M., Grewal H., Bansal M. (2017). Carotid ultrasound for cardiovascular risk prediction: from intima-media thickness to carotid plaques. *Journal of the Indian Academy of Echocardiography & Cardiovascular Imaging*.

[15] Zhang Q., Li C., Han H. (2015). Spatio-temporal quantification of carotid plaque neovascularization on contrast enhanced ultrasound: correlation with visual grading and histopathology. *European Journal of Vascular and Endovascular Surgery*.

[16] Golemati S., Gastounioti A., Nikita K. S. (2013). Toward novel noninvasive and low-cost markers for predicting strokes in asymptomatic carotid atherosclerosis: the role of ultrasound image analysis. *IEEE Transaction on Biomedical Engineering*.

[17] Mitchell C., Korcarz C. E., Gepner A. D. (2018). Ultrasound carotid plaque features, cardiovascular disease risk factors and events: the multi-ethnic study of atherosclerosis. *Atherosclerosis*.

[18] Hyafil F., Schindler A., Sepp D. (2016). High-risk plaque features can be detected in non-stenotic carotid plaques of patients with ischaemic stroke classified as cryptogenic using combined 18F-FDG PET/MR imaging. *European Journal of Nuclear Medicine and Molecular Imaging*.

[19] Spence J. D., Eliasziw M., DiCicco M., Hackam D. G., Galil R., Lohmann T. (2002). Carotid plaque area. *Stroke*.

[20] Landry A., Fenster A. (2002). Theoretical and experimental quantification of carotid plaque volume measurements made by three-dimensional ultrasound using test phantoms. *Medical Physics*.

[21] Chiu B., Egger M., Spence J. D., Parraga G., Fenster A. (2008). Quantification of carotid vessel wall and plaque thickness change using 3D ultrasound images. *Medical Physics*.

[22] Altaf N., Kandiyil N., Hosseini A., Mehta R., MacSweeney S., Auer D. (2014). Risk factors associated with cerebrovascular recurrence in symptomatic carotid disease: a comparative study of carotid plaque morphology, microemboli assessment and the European carotid surgery trial risk model. *Journal of the American Heart Association*.

[23] Sun J., Zhao X. Q., Balu N. (2017). Carotid plaque lipid content and fibrous cap status predict systemic CV outcomes: the MRI substudy in AIM-HIGH. *JACC: Cardiovascular Imaging*.

[24] Van Den Bouwhuijsen Q. J., Bos D., Ikram M. A. (2015). Coexistence of calcification, intraplaque hemorrhage and lipid core within the asymptomatic atherosclerotic carotid plaque: the Rotterdam study. *Cerebrovascular Diseases*.

[25] Huang X., Zhang Y., Meng L. (2018). Identification of ultrasonic echolucent carotid plaques using discrete Fréchet distance between bimodal gamma distributions. *IEEE Transactions on Biomedical Engineering*.

[26] Acharya U. R., Faust O., Alvin A. P. C. (2012). Symptomatic vs. asymptomatic plaque classification in carotid ultrasound. *Journal of Medical Systems*.

[27] Acharya U. R., Krishnan M. M., Sree S. V. (2013). Plaque tissue characterization and classification in ultrasound carotid scans: a paradigm for vascular feature amalgamation. *IEEE Transactions on Instrumentation and Measurement*.

[28] Araki T., Jain P. K., Suri H. S. (2017). Stroke risk stratification and its validation using ultrasonic echolucent carotid wall plaque morphology: a machine learning paradigm. *Computers in Biology and Medicine*.

[29] Wang Y., Wang X., Liu W. (2016). Unsupervised local deep feature for image recognition. *Information Sciences*.

[30] Li J., Yu Z. L., Gu Z., Liu H., Li Y. (2019). MMAN: multi-modality aggregation network for brain segmentation from MR images. *Neurocomputing*.

[31] Lekadir K., Galimzianova A., Betriu À. (2017). A convolutional neural network for automatic characterization of plaque composition in carotid ultrasound. *IEEE Journal of Biomedical and Health Informatics*.

[32] Kats L., Vered M., Zlotogorski-Hurvitz A., Harpaz I. (2019). Atherosclerotic carotid plaque on panoramic radiographs: neural network detection. *International Journal of Computerized Dentistry*.

[33] Jain P., Gupta S., Bhavsar A., Nigam A., Sharma N. (2020). Localization of common carotid artery transverse section in B-mode ultrasound images using faster RCNN: a deep learning approach. *Medical & Biological Engineering & Computing*.

[34] Skandha S. S., Gupta S. K., Saba L. (2020). 3-D optimized classification and characterization artificial intelligence paradigm for cardiovascular/stroke risk stratification using carotid ultrasound-based delineated plaque: Atheromatic™ 2.0. *Computers in Biology and Medicine*.

[35] Fenster A., Blake C., Gyacskov I., Landry A., Spence J. D. (2006). 3D ultrasound analysis of carotid plaque volume and surface morphology. *Ultrasonics*.

[36] Luo X., Yang Y., Cao T., Li Z. (2011). Differences in left and right carotid intima-media thickness and the associated risk factors. *Clinical Radiology*.

[37] He K., Zhang X., Ren S., Sun J. (2015). Spatial pyramid pooling in deep convolutional networks for visual recognition. *IEEE Transactions on Pattern Analysis and Machine Intelligence*.

[38] Ma W., Cheng X., Xu X. (2021). Multilevel strip pooling-based convolutional neural network for the classification of carotid plaque echogenicity. *Computational and Mathematical Methods in Medicine*.

[39] Sanderson C., Paliwal K. K. (2004). Identity verification using speech and face information. *Digital Signal Processing*.

[40] Llewellyn D. J., Lang I. A., Xie J., Huppert F. A., Melzer D., Langa K. M. (2008). Framingham stroke risk profile and poor cognitive function: a population-based study. *BMC Neurology*.

[41] Spence J. D. (2002). Ultrasound measurement of carotid plaque as a surrogate outcome for coronary artery disease. *American Journal of Cardiology*.

[42] Zhou R., Guo F., Azarpazhooh M. R., Spence J. D., Fenster A. (2020). A voxel-based fully convolution network and continuous max-flow for carotid vessel-wall-volume segmentation from 3d ultrasound images. *IEEE Transactions on Medical Imaging*.

[43] Perez L., Wang J. (2017). The effectiveness of data augmentation in image classification using deep learning. https://arxiv.org/abs/1712.04621.

[44] Cheng P. M., Malhi H. S. (2017). Transfer learning with convolutional neural networks for classification of abdominal ultrasound images. *Journal of Digital Imaging*.

[45] Srivastava N., Hinton G., Krizhevsky A., Sutskever I., Salakhutdinov R. (2014). Dropout: a simple way to prevent neuralnetworks from overfitting. *Journal of Machine Learning Research*.

[46] Bottou L., Curtis F. E., Nocedal J. (2018). Optimization methods for large-scale machine learning. *SIAM Review*.

[47] Fawcett T. (2006). An introduction to ROC analysis. *Pattern Recognition Letters*.

[48] Hanley J. A., McNeil B. J. (1982). The meaning and use of the area under a receiver operating characteristic (ROC) curve. *Radiology*.

[49] Bland M. (2015). *An Introduction to Medical Statistics*.

[50] Rosner B. A. (1982). A generalization of the paired t-test. *Journal of Royal Statistical Society*.

[51] Abdi H. (2010). Holm’s sequential Bonferroni procedure. *Encyclopedia of Research Design*.

[52] Ferreira J. A., Zwinderman A. H. (2006). On the Benjamini--Hochberg method. *Annal of Statistics*.

[53] Xie S., Girshick R., Dollár P., Tu Z., He K. Aggregated residual transformations for deep neural networks.

[54] Huang G., Liu Z., Van Der Maaten L., Weinberger K. Q. Densely connected convolutional networks.

[55] Tan M., Le Q. (2019). Efficientnet: rethinking model scaling for convolutional neural networks. *International conference on machine learning*.

[56] Spence J. D. (2006). Technology insight: ultrasound measurement of carotid plaque--patient management, genetic research, and therapy evaluation. *Nature Clinical Practice Neurology*.

[57] Spence J. D., Hackam D. G. (2010). Treating arteries instead of risk factors. *Stroke*.

